# Deep Sequencing Data and Infectivity Assays Indicate that *Chickpea Chlorotic Dwarf Virus* is the Etiological Agent of the “Hard Fruit Syndrome” of Watermelon

**DOI:** 10.3390/v9110311

**Published:** 2017-10-25

**Authors:** Takoua Zaagueri, Laura Miozzi, Monia Mnari-Hattab, Emanuela Noris, Gian Paolo Accotto, Anna Maria Vaira

**Affiliations:** 1 Laboratoire de Biotechnologie Appliquée à l’Agriculture, Institut National de la Recherche Agronomique de Tunisie (INRAT), Université de Carthage, El Rue Hedi Karray Menzah, 1004 Tunis, Tunisia; takoua20@live.fr (T.Z.); mmnari@gmail.com (M.M.-H.); 2 Institute for Sustainable Plant Protection (IPSP), CNR, 10135 Turin, Italy; laura.miozzi@ipsp.cnr.it (L.M.); emanuela.noris@ipsp.cnr.it (E.N.); gianpaolo.accotto@ipsp.cnr.it (G.P.A.)

**Keywords:** watermelon, CpCDV infectious clone, next generation sequencing, mastrevirus, *Geminiviridae*, hard fruit syndrome, *Amalgaviridae*

## Abstract

*Chickpea chlorotic dwarf virus* (CpCDV), a polyphagous mastrevirus, family *Geminiviridae*, has been recently linked to the onset of the “hard fruit syndrome” of watermelon, first described in Tunisia, that makes fruits unmarketable due to the presence of white hard portions in the flesh, chlorotic mottling on the rind, and an unpleasant taste. To investigate the etiological agent of this disease, total RNA extracted from symptomatic watermelon fruits was subjected to small RNA sequencing through next generation sequencing (NGS) techniques. Data obtained showed the presence of CpCDV and two other viral species. However, following validation through polymerase chain reaction (PCR), CpCDV was the only viral species consistently detected in all samples. Watermelon seedlings were then challenged by an agroinfectious CpCDV clone; several plants proved to be CpCDV-infected, and were able to produce fruits. CpCDV infected and replicated in watermelon fruits and leaves, leading to abnormality in fruits and in seed production, similar to those described in field. These results indicate that CpCDV is the etiological agent of the “hard fruit syndrome” of watermelon.

## 1. Introduction

Watermelon (*Citrullus lanatus*) production in the world accounted to about 111 million tons in 2014 (Food and Agriculture Organization Corporate Statistical Database), with China being the top producer. This crop is largely cultivated in tropical and subtropical areas, and is also very popular in the Mediterranean countries, where it is one of the most common and popular summer fruit crops. Watermelon is widely cultivated in the Central and Southern parts of Tunisia, with a yearly production of 510,000 tons in 2014. A hard-fruit syndrome of watermelon (WHFS) has been reported in Tunisia since 1994, with up to 70% incidence in some areas; diseased plants produced unmarketable fruits exhibiting chlorotic mottling on the rind, white hard portions inside the flesh, and an altered taste. In recent years, the incidence has ranged between 10 and 40%, leading growers to switch to alternative crops. WHFS etiology is uncertain up to now, but two begomoviruses (*Tomato yellow leaf curl virus* (TYLCV) and *Tomato yellow leaf curl Sardinia virus*, (TYLCSV)) were recently found associated with this syndrome [1], suggesting a viral etiology for a disease initially attributed to physiological and/or nutritional disorders. Recently, a rolling-circle amplification assay, able to amplify the circular genome of single-stranded (ss) DNA viruses (such as geminiviruses) was performed on fruit samples showing typical symptoms, collected through years in different areas of Tunisia. This led to the unexpected identification of another ssDNA virus, *Chickpea chlorotic dwarf virus*, (CpCDV, genus *Mastrevirus*, family *Geminiviridae*) [2]. This virus was also detected in a high percentage of diseased plants. CpCDV is a polyphagous dicot-infecting mastrevirus, inducing the chickpea stunt disease across North Africa, the Middle East and the Indian subcontinent [3]. Beside chickpea (*Cicer aretinum*), where it was originally described [4], several pulse crops, staple food for millions of people, have been found infected by CpCDV, such as faba bean (*Vicia faba*), lentil (*Lens culinaris*), bean (*Phaseolus vulgaris*) and wild legume species (*Accasia* spp. *Cajanus cajan*, *Dolichus lablab*, *Rinchosia minima*) in Sudan [5]; CpCDV was also detected in *Beta vulgaris* in Iran [6], in papaya (*Carica papaya*) in Burkina Faso [7], in some economically important *Solanaceae* such as pepper (*Capsicum annuum*) in Oman [8], and tomato (*Lycopersicon esculentum*) in Pakistan and Burkina Faso [7,9], in *Malvaceae* as okra (*Abelmoschus esculentus*) in Pakistan [10], and cotton (*Gossypium hirsutum*) in India [11]. CpCDV infects also other cucurbits beside watermelon [2], including squash (*Cucurbita pepo*) in Egypt [12] and cucumber (*Cucumis sativus*) in Pakistan [13]. CpCDV, as with other geminiviruses, is considered to be a dangerous emerging virus, with a high potential of recombination and spread in new areas infecting new crops [14,15]. It is therefore crucial to evaluate its presence and its pathogenicity effects, in order to adopt prevention procedures and limit crop losses. We recently suggested that CpCDV is the etiological agent of WHFS; therefore we investigated this issue in depth on diseased fruits using an NGS approach.

## 2. Materials and Methods

### 2.1 Plant Material

**Figure 1:**
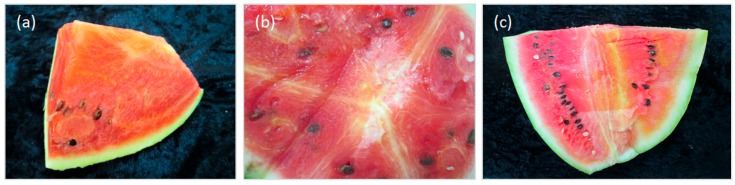
Symptoms on watermelon fruits collected in Tunisian fields used for next generation sequencing (NGS) assays: (**a**) Pa 30/016; (**b**) Pa 44/016; (**c**) Pa 63/016.

Ten watermelon fruits showing different degrees of hardness and discoloration of the flesh, with whitish inserts, deformation of fruits and seeds, and bad taste, were collected in several Tunisian areas (Table 1, Figure 1) from individual plants during the 2016 growing season. Flesh and rind samples were collected and stored at −80 °C.

### 2.2. Nucleic Acid Extraction and Sequencing

For small RNA (sRNA) sequencing, total RNA was extracted from 10 symptomatic fruits using TRIzol Reagent (Thermo Fisher Scientific, Waltham, MA, USA), according to the manufacturer’s instructions. The RNA extracts were individually evaluated using NanoDrop spectrophotometer ND-1000 (Thermo Scientific, Wilmington, DE, USA) and pooled into one sample; three micrograms of the pooled RNA were sent to Human Genetic Foundation sequencing service [16] for library preparation with the TruSeq RNA library Prep Kit v2 (Illumina, San Diego, CA, USA) and sequencing with Illumina NextSeq 500.

Total RNA was also used for complementary DNA (cDNA) synthesis by High-Capacity cDNA Reverse Transcription Kit (Thermo Fisher Scientific, Waltham, MA, USA); total nucleic acid extractions were performed from the same samples, by silica gel-mediated extraction [17].

### 2.3. Small RNA Bioinformatic Analysis

Raw data (Sequence Read Archive database acc. num. SRP119446) were checked for quality reads with FastQC software [18] and Fastx-toolkit [19] was used for removing adapter sequences, low-quality reads and artefacts; sequences shorter than 19 nt and longer than 27 nt were discarded. Reads were then analyzed by the software package VirusDetect [20], using its plant virus database as reference and default parameters. According to this pipeline, reads were assembled in contigs with both a reference-guided and a de novo assembly approach. De novo-assembled contigs were pooled together with those generated from reference-guided assemblies, and then processed to remove redundant sequences. An homology-dependent strategy to identify known and novel virus sequences from the assembled contigs was then employed. Contigs were first compared against reference virus nucleotide sequences using BLASTn and then against the reference virus protein sequences using BLASTx. Contigs matching the same reference sequence were merged to form the final VirusDetect output, and used to derive the coverage of the reference by virus contigs. Based on contigs length and nucleotide identity with the reference viral genome, viral sequences were selected as candidates for validation.

### 2.4. Validation of Candidate Viruses

To confirm the presence of the viral sequences identified in the sRNA library, PCR and reverse transcription PCR (RT-PCR) assays were conducted separately on each of the 10 plants. Specific primers described in literature were used for CpCDV [2], TYLCV and TYLCSV [21], while new primers were designed on de novo-assembled contigs showing similarities to *Watermelon mosaic virus* (WMV) and to amalgaviruses (see Table 2). RNA was used as templates for cDNA synthesis to validate RNA virus infections; total nucleic acids were directly used in PCR to validate DNA virus infections. Four positive controls were used, one for each known virus, and no positive control was obviously available for the new putative amalgavirus, here provisionally named Watermelon amalgavirus 1 (WmAV1); the CpCDV positive control was a DNA extraction from an experimentally-inoculated *N. benthamiana* [2]; for WMV, cDNA synthetized from total RNA extracted from a WMV-infected *N. benthamiana* (isolate 157C, from the IPSP collection) was used; for TYLCV and TYLCSV, DNA extracts from tomato plants experimentally-inoculated with TYLCV (Genbank Acc. No. DQ144621) and TYLCSV (Genbank Acc. No. X61153) agroclones [22] were used. The PCR negative control originated from a non-agroinoculated watermelon fruit obtained from a seed-borne plant (cv. ‘Bontà’). Amplified fragments were analyzed on 1.5% agarose gel in 0.5 X Tris buffer EDTA (TBE).

### 2.5. Agroinfection of Watermelon Seedlings

For the infectivity assays, the agroinfectious clone of the CpCDV Tunisian isolate (TN-Zaghouan-TB2-Watermelon-2015, GenBank accession No. KX580024) [2] was used. This clone was obtained by deleting 455 bp using *Nco*I/*Pst*I from the full length CpCDV genome previously cloned with *Spe*I into pBluescriptKS+, followed by the insertion of a full-length genome in the remaining *Spe*I site. The obtained fragment, corresponding to a ca. 1.8mer of the viral genome, was recovered with *Hind*III/*Sac*I restriction and transferred into the binary vector pBin19, linearized accordingly. The 1.8mer clone was transformed into *Rhizobium radiobacter* (common name *Agrobacterium tumefaciens*, strain LBA4404). Bacteria were grown in liquid YEB/kanamycin/rifampicin medium for 48 h at 28 °C, with shaking, pelleted and resuspended in sterile water. About 30–40 μL of the suspension were inoculated in the stems of watermelon seedlings at first true leaf stage or into the leaf axils of the model plants *Nicotiana benthamiana*, according to described protocols [23].

Several watermelon cultivars for a total of 19 ‘Sugar Baby’, 3 ‘Crimson’, 32 ‘Bontà’, and 61 ‘Sentinel’ plants were tested for infection 28 days post agroinoculation (dpa) by tissue print or PCR. Tissue print positive reactions were always confirmed by dot blotting. Infected watermelon plants were maintained in confined environment in 20cm-diameter pots until fruit delivery. Healthy, non-inoculated watermelon plants were grown as controls in the same conditions. Fruits of about 10 cm in diameter were collected once ripening occurred, and samples from a mixture of flesh and rind were individually taken for DNA extraction.

### 2.6. CpCDV Detection and Replication

Leaf/stem prints (tissue print assay) or total nucleic acids extracts (dot blot assay), fixed on nylon membranes, were used to detect CpCDV. To visualize virus genomic forms, Southern blot assays were performed using total nucleic acids extracted from watermelon leaves and fruits with the TLES buffer-based method (50 mM Tris–HCl, pH 9, 150 mM LiCl, 5 mM EDTA, and 5% SDS), according to [23]. Analyses were conducted on non-infiltrated young stems or leaves, to avoid false positive results owing to *A. tumefaciens* residual presence in the tissue. When PCR was performed for CpCDV detection in experimentally infected watermelon seedlings, primers amplifying a 1300 bp fragment of CpCDV genome (CpCDV-seq1 5′-GTTGCCACCTGCAACGATT-3′ and CpCDV-seq2 5′-CGACACATAAGGTTCAGGTTG-3′) were used. A digoxigenin-labelled probe was synthesized by PCR DIG probe Synthesis kit (Sigma-Aldrich, Darmstadt, Germany) according to manufacturer’s instructions, using the primer pair CpCDV-CP-F/R targeting the coat protein open reading frame (ORF) of the CpCDV Tunisian isolate [2], which amplified a diagnostic 501 bp DNA fragment. The probe was then purified according to instructions and used at 8.4 ng/mL hybridization buffer. The assays were essentially performed as previously described [24].

## 3. Results

### 3.1. Identification of Viral Sequences in the NGS Data

Sequencing resulted in 33,429,123 reads. Once removed low-quality reads and artefacts, 32,765,804 reads were obtained; reads shorter than 19 nt or longer than 27 nt were then discarded, for a total of 20,943,962 remaining reads. Contigs assembly and BLASTn analysis led to the identification of sequences ascribable to CpCDV and *Watermelon mosaic virus* (genus *Potyvirus*, family Potyviridae) (Table 3 and Table S1).

**Figure 2:**
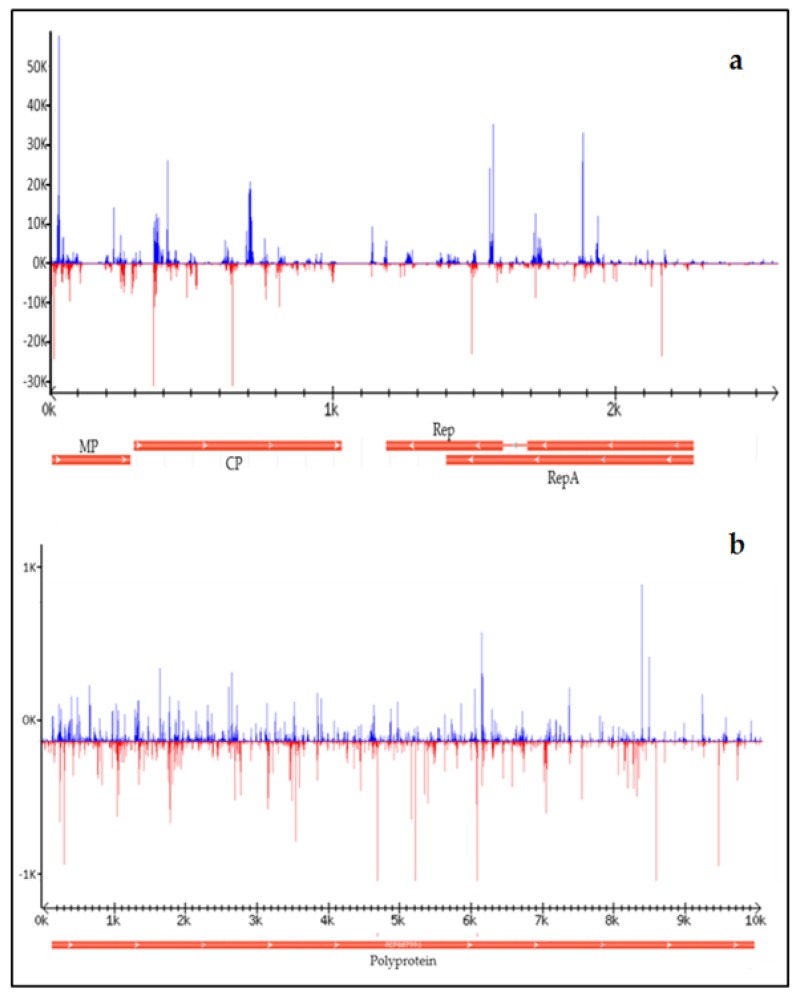
Distribution of sRNAs along (**a**) *Chickpea chlorotic dwarf virus* isolate A Q2510 (GenBank accession No. KC172655) and (**b**) *Watermelon mosaic virus* isolate C05-270 (GenBank accession No. EU660585). Viral genome organization is shown below each graphic; MP: movement protein, CP: capsid protein, Rep: replication protein, RepA: replication protein A.

CpCDV was represented by a total of nine contigs showing high identity (98%) with CpCDV isolate A Q2510 (GenBank accession No. KC172655) with a 100% coverage. About 12% of total reads mapped to the reference sequence of the CpCDV isolate A Q2510, indicating that overall, CpCDV was the most represented virus in the watermelon plants here considered. Examination of sRNA profiles revealed the presence of several hot spots along the viral genome, all located in coding regions (Figure 2a).

WMV was represented by a total of 80 contigs, including a 10,051 nt-long contig that represented the entire viral genome and showed 97% identity with WMV isolate C05-270 (GenBank accession No. EU660585). The high number of contigs obtained for this virus highlights the high variability of reads, probably reflecting the occurrence of sequence variants in the viral populations present in our samples. About 1% of total reads aligned to the full genomic sequence of WMV isolate C05-270. Examination of sRNA profiles revealed a wide distribution of sRNA reads along the whole viral genome (Figure 2b).

Further analyses performed by BLASTx lead to the identification of sequences showing similarity with viruses belonging to different taxa: *Blueberry latent virus* and *Rhododendron virus A* (both genus *Amalgavirus*, family Amalgaviridae), *Ambrosia asymptomatic virus 2* (genus *Badnavirus*, family Caulimoviridae), *Cassava vein mosaic virus* (genus *Cavemovirus*, family Caulimoviridae) (Table 3 and Table S1).

The presence of contigs with an amino acid identity of around 55% with two viruses of the *Amalgavirus* genus suggests that a novel virus belonging to this taxonomic group is present in watermelon. This putative novel amalgavirus is provisionally named *Watermelon amalgavirus 1* (WmAV1). The remaining contigs with amino acid identity to badnaviruses and a cavemoviruses (73% and 43% respectively) showed a reduced length (<250 nt), a low coverage (about 16%), and were therefore excluded from further analysis.

Table S2 provides a list of all contigs obtained from this work showing homology with known viruses according to VirusDetect analysis.

### 3.2. Validation of Viral Sequences by RT-PCR/PCR

**Figure 3:**
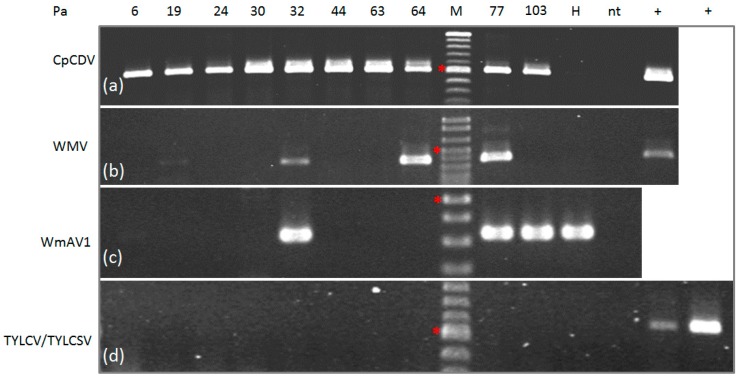
Validation of virus infection in individual watermelon fruit sample used for NGS analysis; polymerase chain reaction (PCR) (**a**,**d**) and reverse transcription PCR (RT-PCR) (**b**,**c**); amplified fragments were separated by electrophoresis in 1.5% agarose gels in 0.5× TBE: (**a**) CpCDV, 501 bp amplified fragment; (**b**) WMV, 379 bp amplified fragment; (**c**) WmAV1, putative novel amalgavirus, 333 bp amplified fragment (no positive control is available); (**d**) TYLCV/TYLCSV, 580 bp amplified fragment, positive controls: TYLCV (+left) and TYLCSV (+right). H, seed-borne watermelon fruit; nt, no template; +, positive controls; M, 100 bp DNA Ladder, (*) indicates the 500 bp fragment.

DNA and RNA extracted from each of the 10 watermelon fruits tested by sRNA NGS were assayed by PCR or RT-PCR, respectively, in order to validate the presence of CpCDV or WMV and the novel amalgavirus WmAV1 in individual samples. CpCDV was the only virus detectable in all samples tested, suggesting its key role in the development of the WHFS. Conversely, WMV was found in 4 out of 10 samples (Pa 19, Pa 32, Pa 64 and Pa 77) and the putative amalgavirus WmAV1 in three out of 10 samples (Pa 32, Pa 77 and Pa 103) (Figure 3). WmAV1 was also detected in the control watermelon plant, cv. Bontà, grown from seed in the glasshouse. This is not surprising, since amalgaviruses are known to be seed transmitted at a very high rate [25].

WHFS has been recently linked to TYLCV and TYLCSV infection [1] and, for this reason, we decided to verify the presence of these viruses in the 10 samples under study, using specific TYLCV/TYLCSV primers. In spite of the frequent detection of the two geminiviruses and CpCDV in mixed infection in watermelon plants collected during past growing seasons, none of the new samples were found to be infected by TYLCV or TYLCSV (Figure 3); this result is in perfect agreement with NGS data, where no TYLCV/TYLCSV-related reads were detected, allowing the involvement of these begomoviruses in this syndrome to be excluded.

### 3.3. CpCDV Sequence Analysis

The BLASTn analysis of the contig representing the full-length CpCDV genome (CONTIG494), showed maximum similarity with the CpCDV KX580024, obtained from watermelon collected in Tunisia in 2015, one year before the collection of the samples used in the present study. The two sequences are 2571 nt long and share a nucleotide identity of 99.1% (2549/2571 nt). The next-closest sequence is a CpCDV isolate from Syria (FR687959) [26], typed as strain A [27,28]. The genome organization is typical of mastreviruses [26].

CONTIG494 contains an unusual nonanucleotide TAATGTTAC in the stem-loop region, different from the canonical geminiviral nonanucleotide TAATATTAC. This non-canonical sequence is also present in CpCDV KX580024, and was confirmed by amplification and sequencing of additional watermelon samples collected in Tunisia in 2015 (2 samples) and 2016 (3 samples). However, all other sequences of CpCDV available in the GenBank repository (more than 200) have a TAATATTAC sequence. To verify whether the non-canonical sequence is correlated with the host plant species infected by CpCDV, we analyzed the two other CpCDV sequences isolated from cucurbits (KF692356 from squash and KT719388 from cucumber) and found that both contained the canonical geminiviral nonanucleotide.

The non-canonical nonanucleotide is uncommon in geminiviruses, but can be found in some chickpea-infecting mastreviruses from Australia, e.g., *Chickpea chlorosis virus* (National Center for Biotechnology Information (NCBI) Reference Sequence NC_014740), *Chickpea chlorosis Australia virus* (NC_022131.1), and *Chickpea redleaf virus* (NC_014739.1) [29,30], as well as in *Dragonfly-associated mastrevirus* (two records), and in one isolate of *Maize streak virus*.

### 3.4. Infectivity Assays

A CpCDV infectious agroclone was used to inoculate watermelon plants in controlled conditions using a tissue print assay to identify infected plants, to be grown for fruit delivery. The infection rate was about 7% (6 out of 83 tested plants), while it reached almost 99% with model plant *N. benthamiana* used as infection controls (222 out of 225 agroinoculated plants). A further experiment was performed, with the hypothesis that the virus concentration could be below the detection limit of the tissue print assay. In this new experiment PCR was used as detection tool, and 18 infected watermelons out of 32 were detected, resulting in a 56% infection rate. A possible explanation is that experimental watermelon infection may in some cases be below tissue print detection threshold, limiting the possibility of routine usage of this widely-used technique for mass diagnosis in these circumstances. Infected watermelon plants showed reduced growth and chlorosis, with smaller and deformed leaves compared to non inoculated plants.

**Figure 4:**
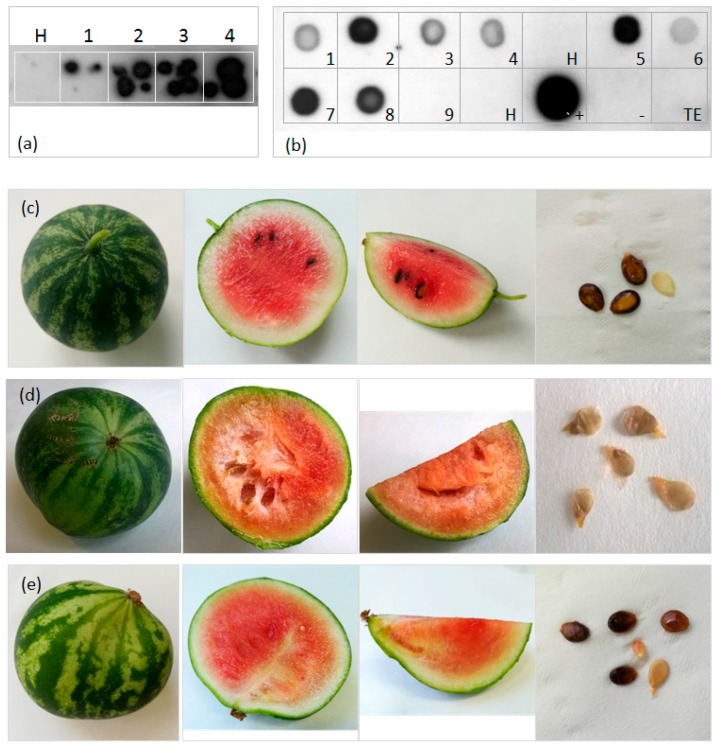
(**a**) Tissue print assay of selected agro-infected watermelon seedlings. Plant and leaf stems are tested, four prints for each plant are performed; H, plant 3/12; 1, plant 4/27; 2, plant 6/14; 3, plant 6/11; 4, plant 6/21. CpCDV CP-specific digoxigenin-labelled probe is used and chemiluminescent detection is performed. (**b**) Dot blot assay of watermelon fruits. Total DNA extracts are prepared using both rind and flesh tissues, 400 ng of DNA/drop, 5 μL volume are spotted on membrane. 1, 2, 3, 4, the four fruits produced by plant 4/27; 5, 6, the two fruits produced by plant 6/11; 7, sole fruit produced by plant 6/14; 8, sole fruit produced by plant 6/21; 9, one of the fruits produced by plant 8/27, agroinoculated but not CpCDV-infected; H, not agro-inoculated watermelon fruit, grown in the same conditions; +, CpCDV-infected *N. benthamiana* leaf tissue; −, healthy *N. benthamiana* leaf tissue; TE, 1x Tris/EDTA buffer, diluent of DNA. (**c**) Not agro-inoculated watermelon fruit cv. ‘Bontà’; (**d**) Symptomatology in watermelon fruit 1 produced by experimentally-infected plant 6/11, cv. ‘Bontà’; (**e**) Symptomatology in watermelon fruit produced by experimentally-infected plant 6/21, cv. ‘Sentinel’; infected fruits show various degree of abnormality in flesh structure with whitish stripes inside the red/orange area, and in seed production.

Four CpCDV-infected watermelon plants (Figure 4a) were kept until fruit delivery. All fruits obtained from CpCDV-infected or healthy watermelons were small in size (10 cm maximum diameter) possibly due to limiting experimental growth conditions: nevertheless they all reached the ripening stage. All the eight fruits collected from the infected plants were positive for virus infection in dot blot assays (see Table 4 and Figure 4b), and showed several degrees of symptoms compared to fruits from healthy plants (Figure 4c), including yellowish/whitish areas or stripes in the flesh, that was discolored (orange instead of red) and, in some cases, displayed a clearly deformed shape. In addition, they all produced few seeds, mainly aborted or immature, especially in the symptomatic parts of the flesh (Figure 4d,e). In five cases, the small fruits showed growth arrest by precocious pod necrosis.

### 3.5. CpCDV Replication

**Figure 5:**
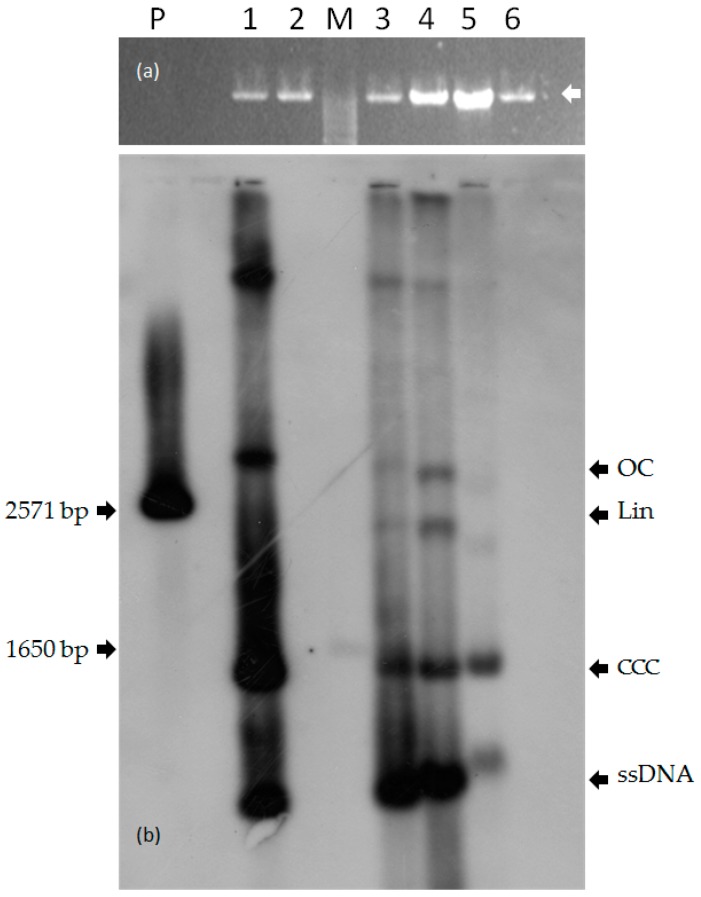
Southern blot assay of DNA extracts of: 1, CpCDV-infected *N. benthamiana*; 2, healthy *N. benthamiana*; 3, CpCDV-infected watermelon plant 6/14 (leaf extract); 4, CpCDV-infected watermelon plant 6/23 (leaf extract); 5, CpCDV-infected watermelon plant 4/27 (fruit extract); 6, healthy watermelon (leaf plus fruit); P, 6 ng of CpCDV genome full length fragment; M, 1 Kb Ladder Plus DNA ladder: the pale 1650 bp band is indicated (Thermo Fisher Scientific, Waltham, MA, USA). On the right, the four CpCDV genome forms found in watermelon leaf and fruit extracts are shown by black arrows; the forms of the double-stranded DNA are indicated: open circular (OC), linear (Lin) and covalently closed circular (CCC), single-stranded DNA is marked (ssDNA) (**a**) ethidium bromide-stained agarose gel showing the high molecular weight genomic DNA bands (white arrow); (**b**) autoradiography film resulting from chemiluminescent detection using CpCDV DIG-labelled probe.

To evaluate the ability of CpCDV to replicate in the aerial parts and in fruits of experimentally inoculated watermelon plants, DNA samples were analyzed by Southern blotting using a CpCDV-specific probe. As it can be seen in Figure 5, both ss and replicative dsDNA forms (in supercoiled and open circular conformations) were detected in watermelon leaves, and also in symptomatic portions of fruits, though at a relatively lower concentration. These viral forms were similar to those detected in the model plant *N. benthamiana*. Conversely, no signal was observed in healthy plants.

This confirms that the CpCDV agroclone obtained from a symptomatic watermelon plant in Tunisia can efficiently replicate in watermelon plants and their fruits, that exhibit symptoms similar to those induced by WHFS.

## 4. Discussion

Over the past years, deep sequencing technologies have opened novel doors to reconstruct viral populations in a high-throughput and cost-effective manner. Up to now, an increasing number of studies have used NGS to either analyze known viruses by means of a reference-guided approach or discover novel viruses using a de novo-based strategy [31]. In this study, the sRNA sequencing of a pool of 10 symptomatic samples was a powerful mean for the identification of CpCDV as the etiological agent of the WHFS and the assembling of its full-length sequence. Indeed, CpCDV was the only virus present in all tested samples, and its agroclone was able to infect watermelon seedlings, actively replicating in the tissues and inducing, in controlled conditions, fruits abnormalities similar to those described for the diseased watermelon plants occurring in open field.

CpCDV, with its wide host range, the seriousness of symptoms induced and its expanding geographical distribution, is an emerging virus and has the potential to become a serious pest for several crops in tropical and Mediterranean countries and worldwide. The two known vectors of CpCDV are *Orosius albicinctus* (Distant) and *Orosius orientalis* (Matsumura), both originally found in Asia. *O. orientalis* was also reported in Tunisia [32] and it is likely that its vector/vectors are already present in other North African countries, and possibly in Southern Europe, posing a new real threat for cucurbits cultivation.

Beside the full-length sequence of the DNA virus CpCDV, the sRNA sequencing has allowed the assembly of the full-length sequence of the RNA virus WMV. WMV infection is widespread in Tunisia on all cucurbit species, at least since the late eighties [33,34]. The virus induces severe green leaf mottling and plant stunting, mainly on melon and squash, both in open field and protected crops, in all major production regions. The virus is less prevalent on watermelon, but when present, it induces mainly leaf symptoms. As a matter of fact, the PCR experiments showed its infection occurred only in a few samples, thus excluding a correlation between this virus and WHFS.

The sRNA sequencing also allowed to identify a previously undescribed virus infecting watermelon, here provisionally named WmAV1, belonging to the *Amalgavirus* genus: as reported for other viral species belonging to the same genus, its infection is apparently symptomless and transmitted through seeds. Amalgaviruses have small dsRNA genomes (about 3.4 kbp) and their virions have not yet been detected or identified; moreover, very little is known about their transmission, apart from seed involvement. The family *Amalgaviridae* is a recently recognized taxon, currently comprising four species of plant-infecting viruses (*Blueberry latent virus*, *Rhododendron virus A*, *Southern tomato virus*, and *Vicia cryptic virus M*) [25,35,36,37,38]. Recently, several new species ascribable to this genus have been described in diverse plant species, following mining transcriptomic data available in public repositories [39]. It is not surprising that amalgavirus-like sequences have been found in watermelon in the present study by analyzing sRNA populations. Considering all these aspects, it is likely that the WmAV1 has a more widespread distribution than expected, but no role in the WHFS.

In summary, this work confirmed that high throughput sequencing analysis of sRNAs is a powerful tool for the identification of both known and new viral sequences and for the definition of new etiological agents. The data obtained by this technique, supported by PCR validation and experimental infection in controlled conditions, allowed the causal link between CpCDV and WHFS to be highlighted for the first time.

## Figures and Tables

**Table 1:** Watermelon fruit samples collected in the 2016 growing season in Tunisia.

Sample	Cultivar	Area of Collection
Pa 6/016	Crimson	Kairouan (Ouled Achour)
Pa 19/016	Crimson	Kairouan (Reggada)
Pa 24/016	Crimson	Kairouan (Reggada)
Pa 30/016	Crimson	Kairouan (Chebika)
Pa 32/016	Crimson	Kairouan (Chebika)
Pa 44/016	Crimson	Kairouan (Sidi Ali Ben Salem)
Pa 63/016	Crimson	Zaghouan (Nadhour)
Pa 64/016	Crimson	Zaghouan (Nadhour)
Pa 77/016	Charleston Gray	Béja (Medjez El Bab)
Pa 103/016	Crimson	Jendouba (Bou Salem)

**Table 2:** Primers used for validation assays, described in literature or designed on sequences of the de novo-assembled contigs. Chickpea chlorotic dwarf virus (CpCDV); Watermelon mosaic virus (WMV); Watermelon amalgavirus 1 (WmAV1), putative novel amalgavirus; Tomato yellow leaf curl virus/Tomato yellow leaf curl Sardinia virus (TYLCV/TYLCSV).

Primers	Sequences	Targeted Virus-Amplicon Size	Targeted Contigs
CpCDV-CP-F/R ^1^	GCAGAATCAAGGGCGAAGAG CGGACCGGGACCATAGTAAG	CpCDV 501 bp	CONTIG494
WMV-CP-F/R	TGATGAGCAGATGGGTGTGA GCTGTTAATTCCCGCGAGAG	WMV 379 bp	CONTIG1352
WmAV1-F/R	TTGCCTGGTCGTGTCTTGAT GCTCAACGATGACAGATGCT	WmAV1 333 bp	CONTIG917
TY1(+)/TY2(-) ^2^	GCCCATGTA(T/C)CG(A/G)AAGCC GG(A/G)TTAGA(A/G)GCATG(A/C)GTAC	TYLCV/TYLCSV 580 bp	-

^1^ [2]; ^2^ [21].

**Table 3:** Viruses identified in the small RNA (sRNA) dataset by VirusDetect analysis; RPKM: reads per kilo base per million mapped reads.

Viral Species	Genus	Genome Length (nt)	No. of Contigs	Contigs Length (Min–Max) (nt)	No. of Reads	RPKM	Type of Analysis
*Chickpea chlorotic dwarf virus* (complete genome)	*Mastrevirus*	2573	9	46–2702	3,197,315	59,331	BLASTn
*Watermelon mosaic virus* (complete genome)	*Potyvirus*	10,051	80	41–10051	402,821	1913	BLASTn
*Blueberry latent virus*/ *Rhododendron virus A* (fusion protein)	*Amalgavirus*	3162/3231	10	53–423	29,626	447/437	BLASTx
*Ambrosia asymptomatic virus 2* (polyprotein)	*Badnavirus*	624	1	134	708	54	BLASTx
*Cassava vein mosaic virus* (ORF3 protein)	*Cavemovirus*	1956	2	103–244	3506	85	BLASTx

**Table 4:** Summary of watermelon fruits assays. Visual interpretation of chemiluminescent reactions data: “+”, slightly positive reaction; “++”, moderately positive reaction; “+++” strongly positive reaction; “−“ negative reaction.

Plant	Cultivar	CpCDV Infection in Plants ^1^	Fruits Tested ^2^	CpCDV Infection in Fruits ^3^
4/27	Sugar Baby	+	1 2 3 4	+ ++ + +
6/11	Bontà	+++	1 ^4^ 2	++ +
6/14	Bontà	+++	1	++
6/21	Sentinel	+++	1 ^5^	++
8/27	Sentinel	−	1	−
Healthy	Bontà	−	1	−

^1^ According to tissue print results. ^2^ All fruits obtained from CpCDV-infected plants were tested; a selection of non-infected plant fruit is presented. ^3^ According to dot blot results. ^4^ Shown in Figure 4d. ^5^ Shown in Figure 4e.

## Supplementary Materials

The following are available online at www.mdpi.com/1999-4915/9/11/311/s1, Table S1: Viral species identified by (a) BLASTn and (b) BLASTx analyses, Table S2: List of all contigs obtained showing homology with known viruses according BLASTn and BLASTx analysis.
