# Supplementary material for: Deep Sequencing Data and Infectivity Assays Indicate that Chickpea Chlorotic Dwarf Virus is the Etiological Agent of the “Hard Fruit Syndrome” of Watermelon
Source: Viruses. 2017 Oct 25;9(11):311. doi: 10.3390/v9110311 (PMC5707518; doi:10.3390/v9110311)
Supplement: Supplementary file 1 [file viruses-09-00311-s001.zip › supplementary files/Table S1.doc]

**Table S1.** Viral species identified by **a**) BLASTn and **b**) BLASTx analyses, using VirusDetect.

a)

| Reference sequence | Length (nt) | Coverage (%) | #Contigs | % Identity | | | Genus | Description |
| --- | --- | --- | --- | --- | --- | --- | --- | --- |
|  |  |  |  | Average | Max | Min |  |  |
| KC172655 | 2573 | 2573 (100) | 8 | 98.12 | 99.03 | 94.44 | Mastrevirus | *Chickpea chlorotic dwarf virus* isolate A Q2510 |
| HE610413 | 2577 | 2469 (95.8) | 3 | 94.05 | 95.45 | 92.93 | Mastrevirus | *Chickpea chlorotic dwarf Pakistan virus* |
| JF273467 | 10032 | 8494 (84.7) | 65 | 94.40 | 100 | 88.36 | Potyvirus | *Watermelon mosaic virus* isolate Cg09-640 |
| EU660583 | 10050 | 8377 (83.4) | 62 | 93.61 | 100 | 89.13 | Potyvirus | *Watermelon mosaic virus* isolate FMF03-141 |
| KP100058 | 10040 | 7655 (76.2) | 55 | 93.66 | 97.35 | 88.00 | Potyvirus | *Watermelon mosaic virus* isolate SangJu6-1 |
| EU660588 | 10045 | 7407 (73.7) | 43 | 92.55 | 97.50 | 88.00 | Potyvirus | *Watermelon mosaic virus* isolate C06-666 |
| EU660580 | 10045 | 7309 (72.8) | 51 | 92.16 | 97.98 | 88.00 | Potyvirus | *Watermelon mosaic virus* isolate CHI87-620 |
| AB369278 | 10037 | 7041 (70.2) | 48 | 93.24 | 100 | 88.00 | Potyvirus | *Watermelon mosaic virus* |
| EU660584 | 10045 | 6995 (69.6) | 41 | 91.76 | 97.56 | 88.11 | Potyvirus | *Watermelon mosaic virus* isolate IR02-54 |
| JX079685 | 10046 | 6928 (69) | 46 | 93.44 | 99.15 | 88.00 | Potyvirus | *Watermelon mosaic virus* strain WMV-ShanXi |
| EU660585 | 10051 | 10051 (100) | 68 | 95.47 | 100 | 87.76 | Potyvirus | *Watermelon mosaic virus* isolate C05-270 |
| EU660586 | 10051 | 10051 (100) | 56 | 93.63 | 100 | 88.00 | Potyvirus | *Watermelon mosaic virus* isolate FBR04-37 |
| JF273469 | 10047 | 10046 (100) | 50 | 93.08 | 98.06 | 88.00 | Potyvirus | *Watermelon mosaic virus* isolate C04-106 |
| HQ384216 | 10057 | 10045 (99.9) | 57 | 93.35 | 97.35 | 87.76 | Potyvirus | *Watermelon mosaic virus* strain Dendrobium |
| KM597070 | 10030 | 10030 (100) | 66 | 93.92 | 100 | 88.36 | Potyvirus | *Watermelon mosaic virus* isolate RKG |

b)

| **Reference sequence** | **Length (aa)** | **Coverage (%)** | **#Contigs** | **% Identity** | | | **Genus** | **Description** |
| --- | --- | --- | --- | --- | --- | --- | --- | --- |
|  |  |  |  | **Average** | **Max** | **Min** |  |  |
| **BAK52524.1** | 1054 | 509 (48.3) | 8 | 54.44 | 66.67 | 35.79 | Amalgavirus | *Blueberry latent virus* fusion protein |
| **ADM36020.1** | 1077 | 396 (36.8) | 7 | 56.82 | 88.24 | 37.76 | Amalgavirus | *Rhododendron virus A* fusion protein |
| **ABY53448.1** | 208 | 34 (16.3) | 1 | 73.53 | 73.53 | 73.53 | Badnavirus | *Ambrosia asymptomatic virus 2* polyprotein |
| **AAB03327.1** | 652 | 108 (16.6) | 2 | 42.73 | 50.00 | 39.74 | Cavemovirus | *Cassava vein mosaic virus* ORF3 protein |
